# Intra‐Individual Variability in Children's Self‐Regulation in Kindergarten Classrooms: An Observational Study

**DOI:** 10.1111/desc.70262

**Published:** 2026-08-10

**Authors:** Nora Tilda Kunz, Niamh Oeri

**Affiliations:** ^1^ Institute for Psychology University of Bern Bern Switzerland

**Keywords:** classroom context, early childhood, intra‐individual variability, multilevel modeling, self‐regulation, observations

## Abstract

Children's ability to self‐regulate varies across days and contextual demands. However, the predominant approach in developmental science is to assess self‐regulation at a single timepoint in one context, limiting our understanding of how and why children's self‐regulation skills vary intra‐ and inter‐individually. The present study addresses this gap by repeatedly observing children's self‐regulation in the classroom 10 times during two different classroom activities. The sample consisted of *N* = 201 Swiss kindergarteners (47.8% female, *M* = 5.4y., *SD* = 0.6). We assessed children's self‐regulation using an observational measure that captures two self‐regulation dimensions: attention control and inhibitory control. To analyze self‐regulation variability, we used multilevel location‐scale models in a dynamic structural equation modeling framework. The findings indicated substantial intra‐individual variability in self‐regulation across the 10 classroom observations. Moreover, children differed significantly in the degree of intra‐individual variability. When controlling for class membership, differences in attention control variability were related to the teacher‐student relationship closeness. Differences in inhibitory control variability were associated with children's gender, with boys showing higher variability than girls. Overall, the present findings indicate that young children's self‐regulation skills can vary substantially across days and classroom activities. These findings emphasize the importance of considering the role of momentary regulatory demands in children's self‐regulation capacities.

## Summary

- Children's self‐regulation was assessed using an observational measure in their everyday classroom context.
- Multilevel location‐scale modeling revealed substantial intra‐individual variability in children's self‐regulation skills.
- Children's self‐regulation skills varied intra‐individually across days and classroom activities.
- Inter‐individual differences in self‐regulation variability were partially related to children's gender and the closeness of the teacher‐student relationship.

## 1 Introduction

Self‐regulation is foundational for various developmental outcomes, including academic achievement and social‐emotional well‐being (Robson et al. 2020). While more than two decades of research document that self‐regulation differences emerge early in life and shape developmental trajectories (e.g., Moffitt et al. 2011; Montroy et al. 2016), more recent empirical evidence indicates substantial intra‐individual variability in children's self‐regulation across days and activities (McCoy et al. 2022; Moschko et al. 2023). Furthermore, these intra‐individual fluctuations seem to be related to both contextual and individual factors (e.g., Holochwost et al. 2023; Munakata and Michaelson 2021). Understanding which factors relate to children's effective regulation on one day and regulatory difficulties in a similar situation on the next day may provide insights into the contributors to these well‐documented inter‐individual self‐regulation differences. To address this gap, the present study examines intra‐individual and inter‐individual variability in kindergarteners’ self‐regulation across two activity types, using repeated classroom observations.

### 1.1 Children's Self‐Regulation

Self‐regulation is the ability to adaptively modulate and control one's emotions, thoughts, attention, and actions in response to momentary demands (e.g., Bailey and Jones 2019; Calkins et al. 2016). It is conceptualized as a multidimensional construct encompassing a range of interrelated regulatory subprocesses (see Jones et al. 2016; Nigg 2017). Evidence from cross‐sectional, longitudinal, and neuroimaging studies indicates that self‐regulation undergoes rapid development during early childhood (Davidson et al. 2006; Montroy et al. 2016; Zelazo et al. 2003). This period is characterized by substantial brain maturation, which occurs in interaction with contextual experiences (Carlson and Wang 2007; Diamond 2013). Early self‐regulatory abilities have been shown to predict a wide range of academic and health outcomes extending into adolescence and adulthood (Edossa et al. 2018; Evans and Rosenbaum 2008; Moffitt et al. 2011). Moreover, individual differences in self‐regulation are observable early in life (Blair and Raver 2015; Montroy et al. 2016; Sawyer et al. 2015). Together, these findings highlight early childhood as a critical period and underscore the importance of identifying factors that shape self‐regulation development.

Because self‐regulation is defined as the ability to adapt to momentary demands (Calkins and Williford 2009), it cannot be considered independent of the context in which it is observed. Accordingly, the capacity to self‐regulate varies as a function of contextual factors. Contextual factors such as classroom routines or social interactions modulate momentary regulatory demands and are thus inextricably linked to self‐regulation (Frick 2025; Zachariou and Whitebread 2022). To account for these contextual demands in the present study, we assess self‐regulation in the classroom using the observational regulation‐related skills measure (RRSM; McCoy et al. 2017, McCoy et al. 2022). The RRSM allows to reliably assess two core dimensions of self‐regulation during typical classroom activities: attention and inhibitory control. These two dimensions have been validated in prior research (Koepp et al. 2022b, 2022a; McCoy et al. 2022). Attention control is the ability to maintain and shift one's attention purposefully while ignoring distractors. Inhibitory control is the ability to inhibit automatic impulses in favor of goals or external expectations (Jones et al. 2016; McCoy et al. 2022). Both dimensions cover constructs from the executive functions and effortful control literature (Bailey and Jones 2019). Accordingly, in the present study we focus on these two core dimensions as they emerge in classroom settings across two different classroom activities.

### 1.2 Intra‐Individual Variability in Children's Self‐Regulation

Intra‐individual variability in self‐regulation refers to fluctuations within an individual across time even within the same or highly similar contexts (Molenaar and Campbell 2009; Wang et al. 2012). In developmental research such intra‐individual fluctuations have often been treated as measurement error rather than as meaningful variation (de Ribaupierre and Lecerf 2018; Van Geert and Van Dijk 2002). An additional important source of such variability arises from differences in contextual demands. Much of the existing literature assesses self‐regulation within a single context at a specific time‐point and subsequently generalizes the findings about individuals’ overall regulatory capacities. As mentioned above, a growing body of research indicates that self‐regulation varies systematically as a function of context. This highlights the importance of incorporating contextual demands systematically into the analysis of self‐regulation.

While several self‐regulation models (Bailey and Jones 2019; Blair and Ku 2022) and developmental theories have acknowledged that children develop in interaction with the contextual systems in which they are embedded (e.g., Bronfenbrenner 1977; Sameroff 2010), the dynamic systems perspective offers a framework for understanding such (context‐dependent) intra‐individual variability in more detail (e.g., Perone et al. 2021; van der Gaag 2023). Dynamic systems theory conceptualizes development as a product of multifaceted, interacting components including neural, cognitive, emotional, motor, and contextual processes to support adaptive, goal‐directed behavior (Spencer et al. 2011; Thelen 2005). From this perspective, self‐regulation is not an isolated ability that children simply apply. Rather self‐regulated behavior emerges from a multicomponent system that is dynamically organized in response to situational demands. For example, a child's ability to regulate behavior in the classroom may reflect the momentary integration of attentional processes, motivational states, prior experiences, and contextual cues such as rules or peer presence. Accordingly, from a dynamic systems perspective, intra‐individual variability in self‐regulation represents a relevant source of information: rather than solely reflecting measurement error, it may reflect the process of establishing adaptive regulatory behaviors in response to various demands. Ultimately, this intra‐individual short‐term variability over moments or days might contribute to developmental growth on a larger timescale such as over months or years (Pérez‐Edgar et al. 2025; Thelen 2005).

Research using reaction time measures to examine intra‐individual variability in preschoolers’ cognitive and attention regulation (Isbell et al. 2018; Judd et al. 2021; Michel et al. 2025) indicates that higher variability is associated with poorer academic performance and lower mean performance in cognitive tasks. Similarly, in a study examining working memory fluctuations in the school context, Dirk and Schmiedek (2016) found that higher day‐to‐day variability in school children's cognitive regulation was associated with lower academic performance and fluid intelligence. Potential explanations for these findings might be that variability reflects limited experience or automatization in learning processes and can therefore be perceived as a vulnerability factor of children at risk for developmental difficulties. Together, these findings highlight that intra‐individual variability can provide critical insights into the quality of inter‐individual differences in children's regulation. However, the presented research predominantly focused on cognitive regulation in standardized tasks, and further evidence is needed on how children regulate behaviors under more naturalistic conditions.

So far, a handful of studies have documented substantial day‐to‐day variability in young children's self‐regulation in the home context (Leonard et al. 2022; Ludwig et al. 2016) and variability across activities in the classroom context (e.g., Eberhart et al. 2024; McCoy et al. 2022). Findings across diverse age groups even suggest that variability in cognitive and attention regulation may be more pronounced in younger individuals (Dirk and Schmiedek 2016; Janvier and Testu 2007; Williams et al. 2005), underscoring the importance of studying this developmental period to gain a more refined understanding of early self‐regulation development.

### 1.3 The Role of Individual and Contextual Factors in Self‐Regulation Variability

From a dynamic‐systems perspective, momentary self‐regulation emerges as a function of a multicomponent system (Perone et al. 2021). In the present study, we evaluated the following factors: individual characteristics (i.e., gender, age), time‐invariant (i.e., relatively stable across time: teacher‐student relationship closeness), and time‐varying contextual factors (i.e., differ across shorter timescales: activity type, peer interaction).

Age and gender are key individual characteristics linked to self‐regulation (e.g., Matthews et al. 2009; Montroy et al. 2016). Previous research has suggested that boys tend to show lower levels of self‐regulation than girls (e.g., DeJoseph et al. 2026; Matthews et al. 2009). However, effects are small and depend on specific task demands (Grissom and Reyes 2019; Usai 2023). Additionally, longitudinal findings suggest potential gender asynchrony in the regulatory development with a slight delay in boys compared to girls (Hosch et al. 2022; Montroy et al. 2016).

Besides individual factors, the literature highlights the classroom as a pivotal developmental context for young children's self‐regulation (Blair and Raver 2015; Koşkulu‐Sancar et al. 2023). From the age of 4, Swiss children typically spend a significant part of their early childhood in kindergarten classrooms. In classrooms the teacher‐student relationship represents a relatively time‐invariant factor associated with children's development. Especially in early childhood classrooms the teacher may function as attachment figures in addition to teaching (Commodari 2013; Hamre and Pianta 2001; Verschueren and Koomen 2012). A close, warm relationship with a teacher can serve as a secure base supporting children's learning (Koşkulu‐Sancar et al. 2023; Spilt et al. 2022) and fostering their capacity to self‐regulate (Blair and Diamond 2008; Cadima et al. 2016). Most studies examining the link between the teacher‐student relationship and children's self‐regulation or executive functions report that a close relationship is associated with a higher regulatory capacity (Cadima et al. 2016; Vandenbroucke et al. 2018). This link seems to be especially evident in children under age six (Koşkulu‐Sancar et al. 2023; Murray et al. 2019; Verschueren and Koomen 2012). Thus, the teacher‐student relationship represents a time‐invariant factor, related to a child's self‐regulation skills. However, most research on teacher‐student relationship closeness has focused on between‐person differences while it remains unknown if the teacher‐student closeness is related to variability in children's self‐regulation across days and different activity types.

Children's momentary self‐regulation is also affected by at least two types of time‐varying contextual factors that characterize typical, recurring routines in kindergarten classrooms (e.g., Timmons et al. 2016; Zachariou and Whitebread 2022): the level of teacher‐ versus self‐direction and presence of peers, in the present study operationalized as activity type and peer interaction. Research from observational studies shows that self‐regulation varies across classroom activities (Eberhart et al. 2024; McCoy et al. 2022). Comparing children's self‐regulation across classroom activities, McCoy et al. (2022) findings suggest that they show higher regulation during transitions than during group work or whole‐class activities. In contrast, Timmons et al. (2016) found that children demonstrated the highest self‐regulation level during small group activities when compared to play, whole group activities, or transitions. Another study examining the role of teacher‐ versus child‐led activities found higher self‐regulation during child‐led activities (Eberhart et al. 2024). In contrast, Moffett et al. (2024) found that children displayed the most inattentive, off‐task behaviors when participating in independent, child‐led activities. To wrap up, the current findings regarding activity type remain mixed, yet they consistently indicate that classroom activities place distinct regulatory demands. Thus, considering them can be crucial for comprehensively understanding self‐regulation and its dynamic nature.

Peer interactions represent another key contextual demand of classrooms (e.g., Finch et al. 2019; Munakata and Michaelson 2021). The mere presence of others can modify momentary regulatory demands (Frick 2025). For example, it can affect children's motivation to self‐regulate in the face of a specific goal (Doebel 2020). Moreover, engaging in peer interactions is demanding as it requires social regulatory capacities, including understanding social cues, perspective taking, or social problem solving (Bailey and Jones 2019; Frick 2025; Sameroff 2010). Thus, since peer interactions can have implications on regulatory demands in a classroom, taking them into account is essential to understanding self‐regulation variability in a comprehensive way.

### 1.4 The Present Study

The present study examined intra‐individual variability in kindergarteners’ self‐regulation. The study was conducted in Swiss kindergartens over five days using the RRSM to repeatedly observe children's self‐regulation skills during two recurring classroom activities: child‐led and teacher‐led activities.

**FIGURE 1:**
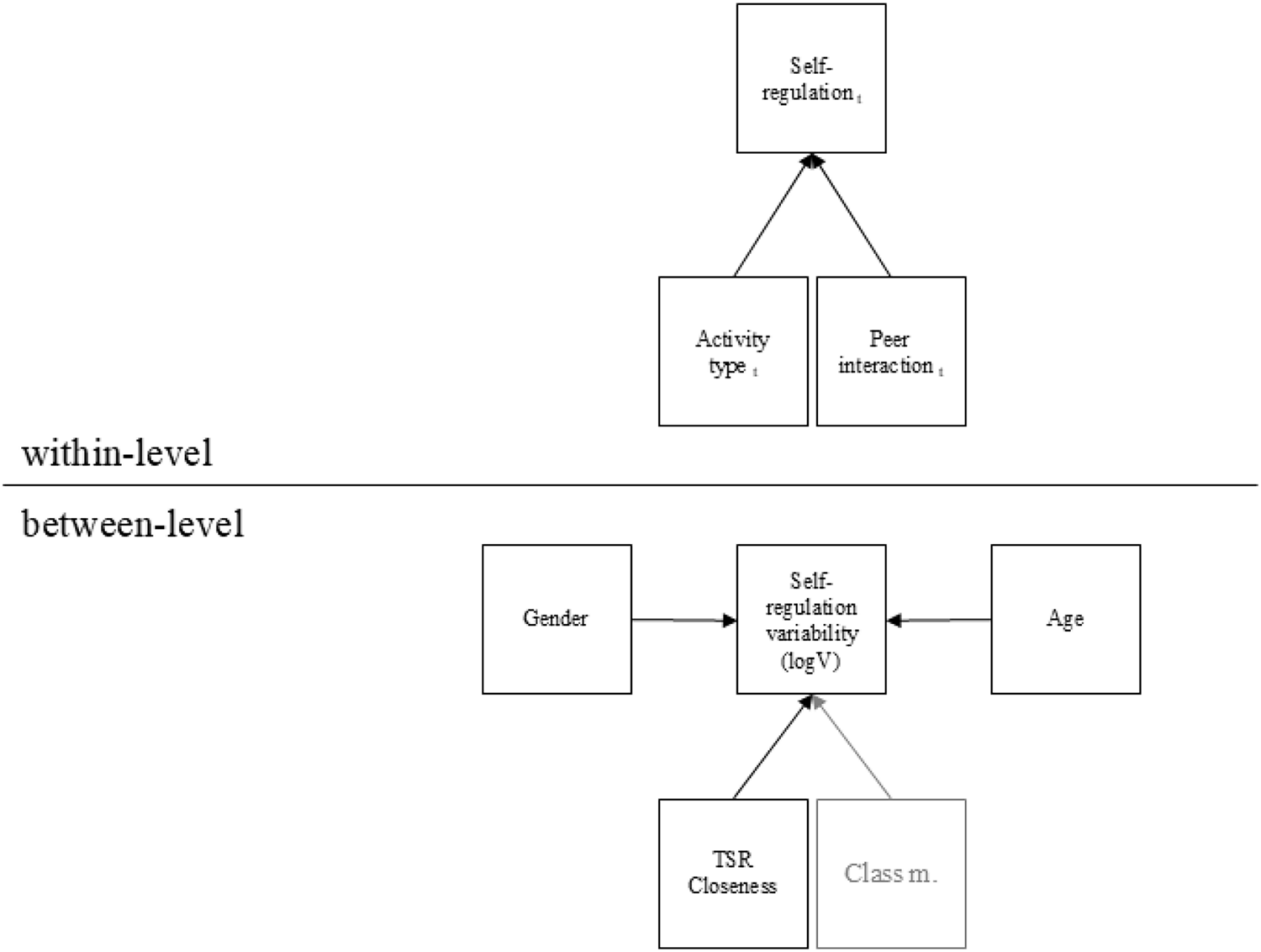
Conceptual Path Diagram for Multilevel Location Scale Models. *Note*: Simplified conceptual path diagram for the multilevel location‐scale model analysis, representing the model with all hypothesized time‐invariant and time‐varying contextual factors and with the individual factors of age and gender. Class m = class membership, LogV = within‐level residual variance, TSR = teacher‐student relationship.

First, we hypothesized that children's self‐regulation skills would significantly vary intra‐individually across repeated observations within individuals (H1). Second, we expected that the degree of intra‐individual variability would vary inter‐individually between children (H2). To evaluate contextual factors that might be associated with intra‐ and inter‐individual variability in children's self‐regulation, we included time‐invariant (i.e., teacher‐student relationship) and time‐varying contextual factors (i.e., activity type and peer interactions). Third, we expected that inter‐individual differences in intra‐individual variability in self‐regulation are associated with the teacher‐student relationship closeness (H3). Fourth, we expected that momentary self‐regulation is related to the time‐varying contextual factors, that is, activity type and peer interaction (H4). Additionally, we included individual characteristics (i.e., age and gender) as covariates to further analyze inter‐individual differences in self‐regulation variability (see Figure 1).

## 2 Methods

### 2.1 Participants

The final sample included *N* = 201 kindergarteners aged between 4.2 and 6.8 years (47.8% female, *M* = 5.4 y., *SD* = 0.6). Initially, 216 children were recruited for the study. However, fifteen children were excluded from the final sample due to developmental delays or neurological disorders (*n* = 7) and absence on more than half of the test dates (*n* = 8). Children were recruited between August and December 2023 from 17 public kindergartens in different (sub)urban areas of the German‐speaking part of Switzerland (see Table S1A). Recruitment was conducted by contacting a randomly selected sample of kindergarten teachers and inviting them to participate. Participation was voluntary, with parents providing informed, written consent, and children their oral assent. In Switzerland, kindergarten constitutes the first two years of the public education system, typically attended by children aged 4 to 6 before they enter first grade. Although it marks the formal entry into schooling and follows the national curriculum, learning is developmentally oriented and play‐based, with a focus on foundational skills for literacy and numeracy as well as fostering self‐regulation and social skills. Children usually attend half‐day sessions in mixed‐age groups. Teachers support children's learning through rich play environments, daily routines, and individualized guidance.

The recruited children were mostly from middle‐class families, reflecting characteristics of the local community. A total of 27% of the children in the sample indicated that (Swiss‐)German was not their mother tongue. Most of the parents reported further educational attainments after finishing compulsory school (95.3%), with two‐thirds of them stating having a (technical) university degree (59.7%).

Ethical approval for the study was provided by the faculty's ethics committee (Approval No. 2023‐07‐01).

### 2.2 Procedure

Trained experimenters visited the kindergartens on five consecutive kindergarten days to collect data on children's self‐regulation within the classrooms. The experimenters followed a standardized observation procedure. On each day, they video‐recorded each child in two classroom activities with distinct regulatory demands: three minutes of teacher‐led whole group activity and three minutes of child‐led semi‐structured play, alone or in smaller groups (i.e., resulting in a total of 10 observations per child). To capture the teacher‐led whole‐group activities, multiple children were filmed simultaneously with static cameras during the morning circle when the group was engaging in routine activities such as book reading or teacher‐led conversations. Thereby, children are expected to remain seated, pay attention to the teacher‐led activity, and raise their hands before speaking. The child‐led semi‐structured play sequences captured participating children one by one during more independent activities alone or in smaller groups, for example, during drawing or completing puzzles. The observed video sequences were later rated by trained coders, following the coding scheme of McCoy and colleagues (2017, 2022). After the five kindergarten visits, parents and teachers answered questionnaires either online or paper‐pencil. The parent questionnaire was available in German, English, French, Spanish, Albanian, Turkish, Croatian, and Portuguese to minimize missing data due to multilingualism in the sample. After the study completion, all teachers received a financial contribution of CHF 200.‐ (approx. EUR 210.‐ or USD 245.‐) to the class fund, and children received a sheet of stickers.

### 2.3 Measures

#### 2.3.1 Dependent Variables of Multilevel Location‐Scale Models

**Self‐Regulation**: The children's self‐regulation in the classroom was assessed using the RRSM (McCoy et al. 2017, McCoy et al. 2022). The observational measure assesses different regulation‐related skills (for example items see Table 1) in the children's classroom context. As described in the procedure section, on each of the five observation days, the experimenter filmed all children twice: once during a teacher‐led whole group activity and once during child‐led semi‐structured play. We excluded participants who were absent on more than half of the test dates (missing observations > 5). All video sequences were coded by trained coders (*N* = 9). The same coder rated all observations available per child. An interrater reliability of Cohen's kappa >0.70 for the training videos was required before coders started coding the study videos. The coding scheme contains 16 items that describe different regulation‐related skills based on behavioral anchors. The items were rated in a two‐step coding process. First, raters decided for each item whether the child “*does*” or “*does not*” demonstrate the behavior‐based skill. Second, raters decided if this skill is observable (i.e., “*does*”) “*consistently*” or “*most of the time*” or if it is not observable (i.e., “*does not*”) “*consistently*” or “*most of the time*” (McCoy et al. 2017, McCoy et al. 2022). Thus, the final scores ranged on a 4‐point pseudo‐Q‐sort scale from (4) ‘*does—consistently*‘ to (1) ‘*does not—consistently’*. Some of the items can be coded with “NA” if no such behavior‐based skill was observable during the three‐minute sequence. Following the procedure of McCoy et al. (2022), we excluded items that could not be coded in more than 50% of the total observations across all children. This resulted in the exclusion of eight items. Moreover, we decided to exclude items if more than 90% of the codings across observations were equal to 4, as this indicated a lack of differentiation between children. Consequently, item 13 was excluded from the analysis too. For a list of the finally included items, see Table 1. To evaluate interrater reliability, 10% of the videos were double‐coded (*n* = 207, Cohen's Kappa unweighted = 0.73; weighted = 0.85). A short version of the coding system is available in the Supporting Information (Table S2A).

**TABLE 1:** Finally included RRSM items and results from the MEFA.

Self‐regulation dimension	Item number	Description of items	Factor loading within		Factor loading between	
			1	2	1	2
Attention control	2	Child pays attention to the activity at hand	**0.93**	0.00	**1.00**	−0.12
Attention control	3	Child can shift attention appropriately within an activity or task	**0.75**	0.15	**0.75**	0.23
Attention control	5	Child can ignore distractions during an activity	**0.93**	−0.04	**0.83**	0.00
Attention control	7	Child shows evidence of listening	**0.52**	0.37	**0.65**	0.35
Inhibitory control	1	Child controls physical movements	0.11	**0.72**	0.08	**0.85**
Inhibitory control	10	Child follows classroom rules and routines independently	0.00	**0.92**	0.01	**0.84**
Inhibitory control	12	Child inhibits inappropriate or automatic responses and enacts appropriate responses	−0.17	**0.86**	−0.13	**1.04**

*Note*: Self‐regulation dimensions with the respective RRSM items that were included in the analysis. Item numbers were derived from the coding manual. The reported factor loadings resulted from the multilevel exploratory factor analysis (MEFA). Factor loadings above 0.40 are in bold.

The final dependent variables for the multilevel location‐scale models were determined based on multilevel exploratory factor analysis (MEFA) with the seven included RRSM items as indicators (Table 1). Two factors emerged, representing attention and inhibitory control. For each of these factors, we computed composite scores by averaging item ratings. Both scales demonstrated good internal consistency (AC α = 0.88, IC α = 0.85).

#### 2.3.2 Time‐Invariant Contextual Factors Multilevel Location‐Scale Models

**Student‐Teacher Relationship Closeness**: Teachers reported on the closeness of the student‐teacher relationship by answering the student‐teacher relationship scale (STRS: Pianta and Nimetz 1991). The 11 items (e.g., I share an affectionate, warm relationship with this child.) were rated on a 5‐point Likert scale ranging from (1) ‘*definitely not true*’ to (5) ‘*definitely true*’. Higher scores indicate a closer relationship. For the variable teacher‐student relationship closeness, a mean score was calculated. The scale demonstrated high internal consistency in the current study (α = 0.81) and in other samples (e.g., Hamre and Pianta 2001).

#### 2.3.3 Time‐Varying Contextual Factors Multilevel Location‐Scale Models

**Contextual Factors**: In addition to the behavioral ratings of the RRSM scale, we also rated the contextual factors of each sequence, that is, activity type and peer interactions. Two distinct activity types were distinguished during the kindergarten observations. The children were either observed during teacher‐led whole‐group activity (i.e., morning circle) or during child‐led semi‐structured play. Activity type was a binary variable, where (0) indicates *teacher‐led activity* and (1) indicates *child‐led activity*. Additionally, the raters of the RRSM documented whether peer interactions (e.g., children played together, shared materials, engaged in cooperation, etc.) occurred during the observed sequence. Peer interaction was a binary variable, where (0) indicates *no interactions*, and (1) indicates that *interactions occurred*.

### 2.4 Statistical Analysis

#### 2.4.1 Data Management

R version 4.3.1 (R Core Team 2020) was used in data preparation and descriptive analysis. There were no exclusions of data points due to outlier identification. Due to single absences and illnesses, not all children could be observed 10 times. Consequently, the RRSM contained some missing observations. The final dataset contained 1906 observations (*n* = 104 missing observations). Not all teachers answered the questionnaire (6.5% missings). Participants with and without missing data on the teacher questionnaire did not differ in terms of their age *t* (199) = −1.58, *p* = 0.12, gender *t* (199) = 0.75, *p* = 0.45, nationality *t* (147) = 1.09, *p* = 0.23, attention control *t* (197) = −1.00, *p* = 0.32, and inhibitory control *t* (199) = 0.58, *p* = 0.56. Consequently, it was assumed that data were missing at random (MAR). In MEFA, missing data are handled with maximum likelihood estimation (ML), which fits the model to all available data (Widaman 2006). For categorical items with few scale points, the weighted least square mean and variance‐adjusted (WLSMV) method was used. The DSEM framework uses a Bayesian approach to handle missing data in the model. This approach has been demonstrated to be robust in dealing with missing data (Hamaker et al. 2018).

#### 2.4.2 Multilevel Exploratory Factor Analysis

To evaluate the factor structure and potential underlying scales of the RRSM items, we conducted MEFA in Mplus (Version 8.10: L. K. Muthén & Muthén 2017). The multilevel approach allows for analyzing factor structures in clustered data (Muthén 1991; Nezlek 2017). Of the total 16 RRSM items, seven (see Table 1) served as indicator variables for the MEFA, clustered within individuals across the measurement occasions (level one: within‐cluster) between different individuals (level two: between‐cluster). Following the recommendation of Van de Vijver and Poortinga (2002) that an intra‐class correlation (ICC) > 0.05 is sufficient to detect the correct number of between‐level factors, the ICCs in our study indicated that the within‐level variation was large enough for meaningful multilevel modeling (0.14< ICCs > 0.32). To interpret the results of the MEFA, we looked at the Eigenvalue plots (Cattell 1966; Kaiser 1960). Additionally, we relied on the root‐mean‐square error of approximation (RMSEA: Steiger 1990), and the Tucker‐Lewis index (TLI: Tucker and Lewis 1973) to evaluate the factor structure, as recommended by Ji et al. (2021). Based on the indicated factor structure (see Figure S1A and Table S3A), we computed composite scores for the resulting self‐regulation dimensions, namely, attention control and inhibitory control.

#### 2.4.3 Multilevel Location‐Scale Models

In the first step, we analyzed the intra‐individual variability in attention and inhibitory control by looking at the ICCs. ICCs represent the proportion of total variance attributable to between‐child differences versus within‐child variability across repeated measurements. Higher ICCs suggest that higher proportions of the variance are attributable to consistent inter‐individual differences, whereas lower ICCs reflect greater intra‐individual variability. Second, we used multilevel location‐scale model in a dynamic structural equation modeling framework (DSEM; Asparouhov et al. 2018) to analyze intra‐individual variability in children's self‐regulation. The DSEM framework allows for combining three types of modeling approaches: First, multilevel modeling, to model the longitudinal data of multiple individuals simultaneously (i.e., nested data); second, structural equation modeling (SEM), to model associations between multiple (latent) variables (e.g., intra‐individual variability and time‐varying and invariant factors); and third, time‐series modeling, to model the lagged effects across the repeated observations (i.e., autoregressive effects) (Asparouhov et al. 2018; McNeish and Hamaker 2020). This allows for simultaneously modeling intra‐individual variability across the 10 observations on the within‐person level and inter‐individual differences in the degree of this variability on the between‐person level. We fitted all models in Mplus (Version 8.10: L. K. Muthén & Muthén 2017) using TYPE = TWOLEVEL RANDOM analysis. We did not model any lagged effects. A Bayesian estimator was used with 3000 iterations and a thinning factor of 10. We have used the default setting in Mplus of two Markov Chain Monte Carlo chains and a burn‐in rate of 50%. Quality of convergence and model fit was determined by the potential scale reduction < 1.05 (Gelman and Rubin 1992) and through visual inspection of posterior trace and autocorrelation plots. To allow the intra‐individual variability to vary across individuals, we estimated multilevel location‐scale models where the within‐person residual variances are modeled to be person‐specific (Jongerling et al. 2015; McNeish and Hamaker 2020). The within‐person residual variance was specified to be a latent variable and was transformed using a log‐linear model. As variances must remain positive, log‐transformation is necessary for the analysis, because it precludes negative values. To interpret the person‐specific residual variances on the original scale, we exponentiated them. We used grand‐mean‐centering for time‐invariant covariates at the between‐level and person‐mean‐centering for the time‐varying covariates at the within‐level (McNeish and Hamaker 2020). To evaluate the multilevel location‐scale model results, we tested the significance of the individual parameters through 95% credible intervals (Asparouhov et al. 2018). Parameters whose 95% credible intervals did not contain zero were considered significantly different from zero. In all models we controlled for the different kindergarten classes. Therefore, we applied a fixed‐effects approach and added 16 dummy‐coded variables reflecting class membership.

As the results of the MEFA indicated a multidimensional structure of self‐regulation, we ran separate models with the two dimensions as the dependent variables. To handle potential convergence problems, models were built stepwise by first adding the covariates separately (Asparouhov and Muthén 2022).

To analyze our first and second hypotheses, we estimated the basic multilevel location‐scale models (McNeish and Hamaker 2020) without covariates. Intra‐individual variability was indicated by the log within‐person residual variance (H1). To evaluate how intra‐individual variability in attention control and inhibitory control differed across individuals (H2), we analyzed the between‐person variance of the log within‐person residual variances.

To address the third hypothesis, the intra‐individual variability in attention and inhibitory control (i.e., log within‐person residual variance) was regressed on the time‐invariant contextual factor of teacher‐student relationship closeness on the between‐level (H3). To answer our fourth hypothesis, attention and inhibitory control were regressed on the time‐varying contextual factors (i.e., activity type, peer interaction) on the within‐level (H4).

Finally, we analyzed the full model, including all covariates, and estimated a model that accounted for individual characteristics. Therefore, we included age and gender as additional covariates at the between‐level. Age was a continuous variable reported in months. Gender was a binary variable (1 = *female*; 0 = *male*). Further, we controlled for weekdays at the within level (1 = *Monday* to 5 = *Friday*).

### 2.5 Transparency and Openness

The present study has been preregistered on the Open Science Framework (https://osf.io/7fc6p/overview). Deidentified data and Mplus output files can be found on the corresponding project on OSF (https://osf.io/qrc9e/overview?view_only=fb2180a3447d4f588d44ed8facae1cda). DeepL and ChatGPT were used to revise text passages for language and style and to refine the R code for data preparation. The authors thoroughly reviewed and edited AI‐suggested content to ensure accuracy and originality.

## 3 Results

Table 2 provides the descriptive statistics and correlation matrix for all variables.

**TABLE 2:** Descriptive statistics and Pearson correlations.

Within‐person level variables		*M* / %	*SD*	Min. / Max.	(1)	(2)	(3)	(4)	
(1) Attention control		3.51	0.51	1.00 / 4.00	—				
(2) Inhibitory control		3.77	0.41	1.00 / 4.00	0.27^*^	—			
(3) Child‐led activity		50.00%	0.50	0 / 1	0.05^*^	0.22^*^	—		
(4) Peer interaction applies		34.77%	0.47	0 / 1	0.06^*^	0.10^*^	0.67^*^	—	

**Between‐person level variables**	***n***	***M* / %**	***SD***	**Min. / Max**.	**(5)**	**(6)**	**(7)**	**(8)**	**(9)**
(5) Attention control	201	3.50	0.27	2.38 / 3.98	—				
(6) Inhibitory control	201	3.77	0.22	2.78 / 4.00	0.39^*^	—			
(7) Teacher‐student relationship closeness	188	4.14	0.69	1.91 / 5.00	0.04	.09	—		
(8) Age^a^	201	65.13	7.49	50.00 / 82.00	0.07	−0.18^*^	0.07	—	
(9) Gender female	201	47.76%	0.51	—	0.06	0.26^*^	0.32^*^	0.02	—

*Note*: Within‐person level *n* = 1906. Estimates with an asterisk (*) are significant.

* *p* < 0.05.

^a^ Age is reported in months.

### 3.1 Preliminary Analysis – Multilevel Exploratory Factor Analysis

To analyze the factor structure of the RRSM items included in the current study, we ran a MEFA. The elbow plot indicated a two‐factor structure (see Figure S1A; scree‐plot test and Eigenvalues > 1; Cattell 1966; Kaiser 1960). Similarly, the TLI = 0.976 and RMSEA = 0.052 supported a two‐factor structure at the within‐ and the between‐levels (see Table S3A). Based on the meaning of the items loading on each factor, we labeled the two factors (i.e., dimensions) as attention control (AC) and inhibitory control (IC). For each dimension, the subsequent models were estimated and reported separately.

### 3.2 Main Analysis – Multilevel Location‐Scale Models

#### 3.2.1 Intra‐Individual Variability

To analyze our first hypothesis regarding intra‐individual variability in self‐regulation, we evaluated the ICCs. In line with the hypothesis, the findings indicated meaningful intra‐individual variability in children's attention and inhibitory control across the 10 observations (AC: *ICC* = 0.18; IC: *ICC* = 0.19). Thus, the ICCs showed that within‐person variability (AC = 82%; IC = 81%) explained a higher percentage of the total variance than between‐person variability (AC: 18%, IC = 19%). Consistent with the ICCs, the multilevel location‐scale model results revealed significant intra‐individual variability in children's attention and inhibitory control across the 10 observations (AC: *Est*. = −1.80, 95% *CI* = −2.19/−1.41; IC: *Est*. = −2.66, 95% *CI* = −3.42/−1.93). As the residual variances were on a log scale, they must be exponentiated to interpret them on the raw scale. On the raw scale, the expected residual variance for attention control was exp(−1.80) = 0.17. For inhibitory control, the expected residual variance on the raw scale was exp(−2.66) = 0.07.

In line with our second hypothesis that children would vary in the degree of intra‐individual variability, the findings of the multilevel location‐scale models showed that children differed significantly in the degree of intra‐individual variability in self‐regulation skills (AC: *Est*. = 0.42, 95% *CI* = 0.30/0.58; IC: *Est*. = 2.42, 95% *CI* = 1.95/3.04; see Table 3). More precisely, assuming normality, 95% of the children's within‐person residual variances for attention control ranged between *e* ^(−1.80± 1.96 √0.42)^ = [0.05 and 0.59] on the raw scale. For inhibitory control, when assuming normality, 95% of the children's within‐person residual variances ranged between *e* ^(−2.66 ± 1.96 √2.42)^ = [0.00 and 1.48] on the raw scale.

**TABLE 3:** Multilevel location‐scale model capturing intra‐ and inter‐individual variability in attention and inhibitory control for the hypotheses 1 and 2.

Model H1 and H2 AC			Model H1 and H2 IC		
Effect	Est	95% CI	Effect	Est.	95% CI
Intercept AC	3.56	[3.52 / 3.59]	Intercept IC	3.85	[3.82 / 3.87]
Intercept LogV	−1.80	[−2.19 / −1.41]	Intercept LogV	−2.66	[−3.42 / −1.93]
LogV on kiga_11	−0.13	[−0.69 / 0.45]	LogV on kiga_11	−0.13	[−1.27 / 1.00]
LogV on kiga_12	−0.00	[−0.52 / 0.55]	LogV on kiga_12	−1.17	[−2.20 / −0.07]
LogV on kiga_13	0.05	[−0.54 / 0.62]	LogV on kiga_13	−0.94	[−2.06 / 0.21]
LogV on kiga_14	−0.77	[−1.32 / −0.20]	LogV on kiga_14	0.15	[−0.96 / 1.23]
LogV on kiga_15	−0.10	[−0.72 / 0.54]	LogV on kiga_15	0.75	[−0.56 / 1.92]
LogV on kiga_16	0.35	[−0.34 / 1.04]	LogV on kiga_16	−0.68	[−1.97 / 0.67]
LogV on kiga_17	0.24	[−0.23 / 0.78]	LogV on kiga_17	0.62	[−0.50 / 1.71]
LogV on kiga_18	0.17	[−0.42 / 0.75]	LogV on kiga_18	0.23	[−0.93 / 1.37]
LogV on kiga_19	0.08	[−0.56 / 0.73]	LogV on kiga_19	0.05	[−1.17 / 1.34]
LogV on kiga_20	0.06	[−0.54 / 0.70]	LogV on kiga_20	0.81	[−0.47 / 2.04]
LogV on kiga_21	−0.18	[−0.79 / 0.49]	LogV on kiga_21	−1.78	[−3.00 / −0.55]
LogV on kiga_22	−0.34	[−1.04 / 0.38]	LogV on kiga_22	−0.19	[−1.62 / 1.19]
LogV on kiga_23	0.92	[0.32 / 1.51]	LogV on kiga_23	−0.16	[−1.30 / 1.01]
LogV on kiga_24	0.09	[−0.64 / 0.83]	LogV on kiga_24	0.90	[−0.55 / 2.32]
LogV on kiga_25	1.27	[0.53 / 1.97]	LogV on kiga_25	1.02	[−0.35 / 2.43]
LogV on kiga_26	−0.37	[−1.00 / 0.26]	LogV on kiga_26	0.12	[−1.16 / 1.38]
Variance AC	0.03	[0.02 / 0.04]	Variance IC	0.01	[0.01 / 0.02]
Variance LogV	0.42	[0.30 / 0.58]	Variance LogV	2.42	[1.95 / 3.04]
R^2^ between	35.3%			22.8%	

*Note*: Estimates (Unstandardized posterior median) and 95% credible intervals (CI) for the basic multilevel location‐scale models without covariates, with attention control (AC) and inhibitory control (IC) as the dependent variables. Kiga_11 to kiga_26 are dummy‐coded variables representing class membership at the between‐level. Nb. of observations = 1906.

#### 3.2.2 Understanding Intra‐Individual Variability – Analyzing the Role of Contextual and Individual Factors

To examine if contextual factors are related to children's intra‐individual variability in self‐regulation, we included covariates at the between‐ and the within‐level of the models.

To address the third hypothesis that differences in the degree of intra‐individual variability in self‐regulation are associated with the time‐invariant contextual factor (i.e., teacher‐student relationship closeness), we included relationship closeness on the between‐level of the models. When controlling for class membership using a fixed‐effects approach, we found a significant association between the log within‐person residual variance of attention control and relationship closeness (AC: *Est*. = −0.33, 95% CI = −0.56/−0.11). We found no significant association for inhibitory control (IC: *Est*. = −0.15, 95% CI = −0.60/0.28; see Table 4). Thus, partly consistent with our hypothesis (H3), intra‐individual variability in attention control was related to the teacher‐student relationship closeness when controlling for class membership.

**TABLE 4:** Multilevel location‐scale model with the time‐invariant covariate TSRC for attention and inhibitory control for the hypothesis 3.

Model H3 AC			Model H3 IC		
Effect	Est	95% CI	Effect	Est.	95% CI
Intercept AC	3.56	[3.52 / 3.59]	Intercept IC	3.85	[3.82 / 3.87]
Intercept TSR closeness	0.00	[−0.09 / 0.11]	Intercept TSR closeness	0.01	[−0.10 / 0.10]
Intercept LogV	−1.75	[−2.11 / −1.36]	Intercept LogV	−2.64	[−3.38 / −1.88]
LogV on TSR closeness	−0.33	[−0.56 / −0.11]	LogV on TSR closeness	−0.15	[−0.60 / 0.28]
LogV on kiga_11	−0.57	[−1.20 / 0.07]	LogV on kiga_11	−0.37	[−1.65 / 0.93]
LogV on kiga_12	0.03	[−0.50 / 0.56]	LogV on kiga_12	−1.17	[−2.23 / −0.07]
LogV on kiga_13	−0.01	[−0.58 / 0.58]	LogV on kiga_13	−0.97	[−2.14 / 0.18]
LogV on kiga_14	−0.84	[−1.41 / −0.28]	LogV on kiga_14	0.12	[−1.00 / 1.22]
LogV on kiga_15	−0.36	[−0.99 / 0.30]	LogV on kiga_15	0.62	[−0.68 / 1.98]
LogV on kiga_16	0.28	[−0.37 / 0.96]	LogV on kiga_16	−0.70	[−2.00 / 0.66]
LogV on kiga_17	0.30	[−0.24 / 0.83]	LogV on kiga_17	0.66	[−0.42 / 1.67]
LogV on kiga_18	0.09	[−0.47 / 0.66]	LogV on kiga_18	0.19	[−0.99 / 1.33]
LogV on kiga_19	0.13	[−0.51 / 0.76]	LogV on kiga_19	0.10	[−1.22 / 1.35]
LogV on kiga_20	0.02	[−0.63 / 0.61]	LogV on kiga_20	0.78	[−0.50 / 2.01]
LogV on kiga_21	−0.10	[−0.71 / 0.54]	LogV on kiga_21	−1.79	[−3.00 / −0.53]
LogV on kiga_22	−0.48	[−1.18 / 0.18]	LogV on kiga_22	−0.25	[−1.68 / 1.12]
LogV on kiga_23	0.98	[0.41 / 1.55]	LogV on kiga_23	−0.14	[−1.31 / 1.02]
LogV on kiga_24	0.03	[−0.68 / 0.72]	LogV on kiga_24	0.85	[−0.53 / 2.21]
LogV on kiga_25	1.06	[0.35 / 1.78]	LogV on kiga_25	0.93	[−0.46 / 2.40]
LogV on kiga_26	−0.38	[−1.03 / 0.25]	LogV on kiga_26	0.09	[−1.19 / 1.34]
Variance AC	0.03	[0.02 / 0.04]	Variance IC	0.01	[0.01 / 0.02]
Variance TSR closeness	0.48	[0.40/ 0.60]	Variance TSR closeness	0.48	[0.39 / 0.59]
Variance LogV	0.39	[0.27 / 0.54]	Variance LogV	2.44	[1.95 / 3.05]
R^2^ between	45.1%			23.5%	

*Note*: Estimates (Unstandardized posterior median) and 95% credible intervals (CI) for the multilevel location‐scale models with the within‐person residual variance in attention control (AC) and inhibitory control (IC) as the dependent variables and with time‐invariant covariate teacher‐student relationship closeness (TSRC). Kiga_11 to kiga_26 are dummy‐coded variables representing class membership. Nb. of observations = 2010.

To address the fourth hypothesis that momentary self‐regulation is related to the time‐varying contextual factors (H4), we included activity type and peer interaction as covariates on the within‐levels of the models. In line with our expectation, the results indicated a significant relation between the activity type and attention control and between activity type and inhibitory control although effects were small (AC: *Est*. = −0.09, 95% *CI* = −0.15/−0.04; IC: *Est*. = 0.03, 95% *CI* = 0.01/0.04; see Table 5). More precisely, the results indicated that children showed higher attention control during the teacher‐led compared to the child‐led activities. Regarding inhibitory control, the results indicated that children showed higher control during child‐led compared to teacher‐led activities. Other than expected, no significant differences were found regarding peer interaction.

**TABLE 5:** Multilevel location‐scale models with the time‐varying covariates activity type and peer interaction for attention and inhibitory control for the hypothesis 4.

Model H4 AC			Model H4 IC		
Effect	Est	95% CI	Effect	Est.	95% CI
Intercept AC	3.56	[3.53 / 3.59]	Intercept IC	3.84	[3.82 / 3.87]
Intercept activity type	0.51	[0.48 / 0.53]	Intercept activity type	0.51	[0.48 / 0.53]
Intercept peer interaction	0.37	[0.34/ 0.39]	Intercept peer interaction	0.37	[0.34 / 0.39]
Intercept LogV	−1.80	[−2.20 / −1.41]	Intercept LogV	−2.69	[−3.45 / −1.93]
AC on activity type	−0.09	[−0.15 / −0.04]	IC on activity type	0.03	[0.01 / 0.04]
AC on peer interaction	0.05	[−0.01 / 0.10]	IC on peer interaction	−0.01	[−0.02 / 0.01]
LogV on kiga_11	−0.13	[−0.72 / 0.46]	LogV on kiga_11	−0.14	[−1.28 / 1.02]
LogV on kiga_12	−0.05	[−0.60 / 0.51]	LogV on kiga_12	−1.15	[−2.21 / −0.08]
LogV on kiga_13	0.06	[−0.57 / 0.67]	LogV on kiga_13	−0.93	[−2.08 / 0.26]
LogV on kiga_14	−0.79	[−1.37 / −0.21]	LogV on kiga_14	0.19	[−0.90 / 1.30]
LogV on kiga_15	−0.06	[−0.73 / 0.61]	LogV on kiga_15	0.76	[−0.54 / 2.03]
LogV on kiga_16	0.34	[−0.31 / 1.03]	LogV on kiga_16	−0.65	[−1.93 / 0.64]
LogV on kiga_17	0.28	[−0.26 / 0.83]	LogV on kiga_17	0.65	[−0.43 / 1.73]
LogV on kiga_18	0.19	[−0.43 / 0.77]	LogV on kiga_18	0.26	[−0.93 / 1.39]
LogV on kiga_19	−0.05	[−0.70 / 0.66]	LogV on kiga_19	0.08	[−1.19 / 1.44]
LogV on kiga_20	0.03	[−0.61 / 0.71]	LogV on kiga_20	0.78	[−0.47 / 2.05]
LogV on kiga_21	−0.21	[−0.87 / 0.46]	LogV on kiga_21	−1.78	[−3.01 / −0.46]
LogV on kiga_22	−0.36	[−1.08 / 0.32]	LogV on kiga_22	−0.20	[−1.59 / 1.10]
LogV on kiga_23	0.97	[0.35 / 1.55]	LogV on kiga_23	−0.15	[−1.37 / 1.01]
LogV on kiga_24	0.04	[−0.68 / 0.76]	LogV on kiga_24	0.89	[−0.56 / 2.25]
LogV on kiga_25	1.31	[0.59 / 2.06]	LogV on kiga_25	1.00	[−0.40 / 2.42]
LogV on kiga_26	−0.39	[−1.01 / 0.28]	LogV on kiga_26	0.12	[−1.12 / 1.38]
Variance AC	0.03	[0.02 / 0.04]	Variance IC	0.01	[0.01 / 0.02]
Variance activity type	0.00	[0.00 / 0.00]	Variance activity type	0.00	[0.00 / 0.00]
Variance peer interaction	0.01	[0.01 / 0.02]	Variance peer interaction	0.01	[0.01 / 0.02]
Variance LogV	0.45	[0.32 / 0.61]	Variance LogV	2.39	[1.93 / 3.00]
R^2^ within	1.0%			0.9%	
R^2^ between	35.8%			22.9%	

*Note*. Estimates (Unstandardized posterior median) and 95% credible intervals (CI) for the multilevel location‐scale models with attention control (AC) and inhibitory control (IC) as the dependent variables and with time‐varying covariates activity type and peer interaction. Kiga_11 to kiga_26 are dummy‐coded variables representing class membership. Nb. of observations = 1906.

For attention control and inhibitory control, all results remained the same when the full models, including time‐invariant and time‐varying contextual factors at once, were estimated (see Table S5A).

When adding the individual factors as covariates in the models, results revealed significant age differences (AC: *Est*. = −0.02, 95% CI = −0.04/−0.01): younger children displayed more variability in attention control. In the inhibitory control model, age was unrelated to intra‐individual variability. In addition, no gender differences were found for attention control. In contrast, results revealed significant gender differences regarding intra‐individual variability in inhibitory control: boys showed higher variability in inhibitory control compared to girls (IC: *Est*. = −1.00, 95% CI = −1.50/−0.52). Weekday was unrelated to children's attention and inhibitory control. The detailed results are reported in Tables 6, and S6A in the Supporting Information. Path diagrams visualizing the standardized results are presented in Figures S2A and S3A.

**TABLE 6:** Multilevel location‐scale models with all covariates for attention and inhibitory control.

Model with covariates AC			Model with covariates IC		
Effect	Est	95% CI	Effect	Est.	95% CI
Intercept AC	3.56	[3.52 / 3.59]	Intercept IC	3.85	[3.82 / 3.87]
Intercept activity type	0.51	[0.48 / 0.53]	Intercept activity type	0.51	[0.48 / 0.53]
Intercept peer interaction	0.37	[0.34 / 0.39]	Intercept peer interaction	0.37	[0.34 / 0.39]
Intercept weekday	2.90	[2.83 / 2.98]	Intercept weekday	2.90	[2.83 / 2.98]
Intercept TSR closeness	−0.00	[−0.11 / 0.10]	Intercept TSR closeness	−0.00	[−0.11 / 0.10]
Intercept age	−0.01	[−1.09 / 1.04]	Intercept age	−0.01	[−1.09 / 1.04]
Intercept gender	0.49	[0.41 / 0.56]	Intercept gender	0.49	[0.42 / 0.56]
Intercept LogV	−1.70	[−2.09 / −1.29]	Intercept LogV	−2.16	[−2.94 / −1.38]
AC on activity type	−0.09	[−0.14 / −0.04]	IC on activity type	0.03	[0.01 / 0.04]
AC on peer interaction	0.04	[−0.01 / 0.10]	IC on peer interaction	−0.01	[−0.02 / 0.01]
AC on weekday	0.01	[−0.00 / 0.02]	IC on weekday	−0.00	[−0.01 / 0.00]
LogV on TSR closeness	−0.31	[−0.54 / −0.07]	LogV on TSR closeness	0.22	[−0.25 / 0.66]
LogV on age	−0.02	[−0.04 / −0.01]	LogV on age	0.00	[−0.03 / 0.03]
LogV on gender	−0.08	[−0.33 / 0.17]	LogV on gender	−1.00	[−1.50 / −0.52]
LogV on kiga_11	−0.60	[−1.24 / 0.08]	LogV on kiga_11	0.04	[−1.23 / 1.36]
LogV on kiga_12	−0.02	[−0.54 / 0.52]	LogV on kiga_12	−1.22	[−2.27 / −0.24]
LogV on kiga_13	−0.04	[−0.63 / 0.52]	LogV on kiga_13	−0.80	[−1.91 / 0.27]
LogV on kiga_14	−0.85	[−1.41 / −0.28]	LogV on kiga_14	0.17	[−0.98 / 1.19]
LogV on kiga_15	−0.32	[−0.94 / 0.36]	LogV on kiga_15	0.82	[−0.44 / 2.11]
LogV on kiga_16	0.21	[−0.45 / 0.87]	LogV on kiga_16	−0.84	[−2.07 / 0.42]
LogV on kiga_17	0.31	[−0.25 / 0.86]	LogV on kiga_17	0.47	[−0.63 / 1.53]
LogV on kiga_18	0.11	[−0.47 / 0.69]	LogV on kiga_18	0.33	[−0.80 / 1.42]
LogV on kiga_19	−0.02	[−0.69 / 0.63]	LogV on kiga_19	−0.06	[−1.33 / 1.20]
LogV on kiga_20	−0.03	[−0.67 / 0.61]	LogV on kiga_20	0.87	[−0.35 / 2.11]
LogV on kiga_21	−0.17	[−0.83 / 0.49]	LogV on kiga_21	−1.97	[−3.29 / −0.77]
LogV on kiga_22	−0.53	[−1.22 / 0.13]	LogV on kiga_22	−0.41	[−1.72 / 0.88]
LogV on kiga_23	1.06	[0.48 / 1.65]	LogV on kiga_23	−0.10	[−1.23 / 1.02]
LogV on kiga_24	0.05	[−0.68 / 0.79]	LogV on kiga_24	0.77	[−0.65 / 2.15]
LogV on kiga_25	1.23	[0.48 / 1.99]	LogV on kiga_25	0.84	[−0.61 / 2.26]
LogV on kiga_26	−0.39	[−1.02 / 0.22]	LogV on kiga_26	−0.06	[−1.31 / 1.18]
Variance AC	0.03	[0.02 / 0.04]	Variance IC	0.01	[0.01 / 0.02]
Variance activity type	0.00	[0.00 / 0.00]	Variance activity type	0.00	[0.00 / 0.00]
Variance peer interaction	0.01	[0.01 / 0.02]	Variance peer interaction	0.01	[0.01 / 0.02]
Variance weekday	0.06	[0.02 / 0.13]	Variance weekday	0.06	[0.02 / 0.13]
Variance TSR closeness	0.51	[0.42 / 0.63]	Variance TSR closeness	0.51	[0.41 / 0.63]
Variance age	59.97	[49.58 / 74.39]	Variance age	60.08	[49.64 / 74.28]
Variance gender	0.28	[0.23 / 0.35]	Variance gender	0.28	[0.23 / 0.35]
Variance LogV	0.39	[0.27 / 0.56]	Variance LogV	2.22	[1.78 / 2.79]
R^2^ within	1.2%			0.9%	
R^2^ between	49.7%			32.3%	

*Note*: Estimates (Unstandardized posterior median) and 95% credible intervals (CI) for the multilevel location‐scale models with attention control (AC), inhibitory control (IC), and their within‐person residual variances as dependent variables and with the time‐invariant teacher‐student relationship closeness, and time‐varying covariates activity type, and peer interaction. Covariates were: weekday at the within‐level; age and gender at the between‐level. Kiga_11 to kiga_26 are dummy‐coded variables representing class membership at the between‐level. Nb. of observations = 1906.

### 3.3 Subsequent Exploratory Analysis

To better understand the quality of the intra‐individual variability and the role of activity type, we ran the basic multilevel location‐scale models separately for the two activity types. Thereby, we aimed to understand if intra‐individual variability also occurred day‐to‐day across multiple observations of the same activity type. Thus, for both dimensions, that is, attention and inhibitory control, we ran the model with the five teacher‐led activities and the five child‐led activities. Similar to the main analysis, exploratory analyses for the separate activity types indicated substantial intra‐individual variability in attention and inhibitory control. The detailed results are presented in Table S4A in the Supporting Information.

In line with the multilevel location‐scale model results, the ICCs indicated substantial intra‐individual variability. Children showed less day‐to‐day intra‐individual variability during teacher‐led than child‐led activities. In the teacher‐led activities, 49% of the variance in attention control was explained by within‐person variability (ICC = 0.51), and 68% of the variance in inhibitory control was explained by within‐person variability (ICC = 0.32). In the child‐led activities, 80% of the variance in attention control was explained by within‐person variability (ICC = 0.20), and 83% of the variance in inhibitory control was explained by within‐person variability (ICC = 0.17).

## 4 Discussion

The present study examined intra‐ and inter‐individual variability in kindergarteners' self‐regulation skills across multiple observations of two recurring classroom activities. Substantial intra‐individual variability was found for both self‐regulation dimensions (attention and inhibitory control) and across days and activity types. Moreover, the results showed significant inter‐individual differences in the degree of intra‐individual variability, suggesting that some children exhibit greater fluctuations in self‐regulation than others. These differences were partially associated with the teacher‐student relationship closeness, as well as with age and gender.

### 4.1 Variability in Children's Self‐Regulation in the Classroom

In line with our first hypothesis (Leonard et al. 2022; Ludwig et al. 2016), we found that kindergartners’ attention and inhibitory control varied intra‐individually across 10 classroom observations. The ICC results indicated that a higher percentage of the overall variance was explained by within‐person than by between‐person differences. These findings contribute to prior work demonstrating that young children's self‐regulation varies substantially in everyday contexts (Ludwig et al. 2016; McCoy et al. 2022). Existing research on intra‐individual variability in young children's self‐regulation has primarily focused on day‐to‐day variability in the home context (Ludwig et al. 2016) or on variability across activity types in the classroom context (e.g., Eberhart et al. 2024; McCoy et al. 2022). The present study extends these findings by examining both intra‐individual variability across days and activity types in in children's everyday classroom context.

In line with our second hypothesis, our results indicated that children differ inter‐individually in the degree of intra‐individual variability in self‐regulation. Specifically, our study demonstrated that some children showed higher fluctuations across the 10 observations, while others showed more stable self‐regulation skills. Accordingly, the degree to which children's self‐regulation varies across everyday situations might be a relevant factor driving early individual differences. This finding adds to recent research revealing substantial individual differences in intra‐individual variability across more fine‐grained timescales (i.e., response time variability across trials) in standardized cognitive tasks (Judd et al. 2024). Additionally, our findings indicate that such differences in variability are also observable in children's everyday contexts and thereby provide unique insights into how regulation contributes to individual functioning in applied contexts.

### 4.2 The Role of Contextual and Individual Factors for Variability in Self‐Regulation

Existing literature has demonstrated associations between children's regulation‐related skills and teacher‐student relationship closeness at the between‐person level (Cadima et al. 2016; Vandenbroucke et al. 2018). Additionally, momentary self‐regulation was linked to teacher‐support at the within‐person level (Blume and Schmiedek 2024). Accordingly, we expected the teacher‐student relationship closeness to be related to intra‐individual variability in self‐regulation. In the present study, variability in self‐regulation was only related to the relationship closeness when controlling for class membership. Although effects were small, the findings indicated that children with closer teacher relationships tended to show less variability in attention control, when accounting for general differences between classrooms (e.g., response biases; order and routines). These results support the vital role of a close relationship with teachers for children's capacity to consistently self‐regulate across demands. However, it is important to note that closeness represents only one dimension of teacher‐student relationship. Other relevant dimensions, such as degree of conflict or autonomy (Koşkulu‐Sancar et al. 2023), were not assessed in the present study. For a comprehensive understanding, future research should examine how multiple relationship dimensions relate to intra‐individual variability.

Consistent with previous findings (Eberhart et al. 2024; McCoy et al. 2022; Timmons et al. 2016) and the fourth hypothesis, we found that children's self‐regulation varied across activity types. Although, effects were small and therefore must be interpreted with caution, the present findings indicate that children showed higher attention control during teacher‐led than child‐led activities. This aligns with findings of Moffett et al. (2024), who showed that children displayed more off‐task behavior (i.e., inattention) during independent child‐led activities than during teacher‐led routine activities. A possible reason for higher attention control during teacher‐led activities could be the stronger teacher guidance of children's attention (Barnes et al. 2022; Martin et al. 2024). Additionally, it might be easier to regulate attention as there are usually fewer distractors in the classroom while everyone is in the circle.

In contrast, children showed lower inhibitory control during teacher‐led than child‐led activities. One explanation could be that the activities put different regulatory demands upon children's inhibitory control (Doebel 2020). Doebel pointed out that children's inhibitory control may vary in response to the meaningfulness of an activity's goal. Child‐led activities allow children to pursue self‐guided activities that are meaningful to them (Baker et al. 2023; Stefanou et al. 2004). In contrast, teacher‐led activities often involve rules and goals set by teachers, which may be less meaningful to children (Boekaerts & Niemivirta, 2000). Additionally, the teacher‐led activities may place higher inhibitory demands, due to social expectations (e.g., sitting still without distracting neighbors, raising hands before talking). In sum, our results emphasize the importance of assessing self‐regulation while accounting for the activity that sets specific regulatory demands. However, as most prior studies examined distinct activity types, direct comparisons are limited, highlighting the need for more systematic research addressing this gap.

In subsequent exploratory analyses, we examined if day‐to‐day variability also occurred across multiple observations within the same activity type. Our results showed that when considering only variability within teacher‐led activities, within‐person differences accounted for a smaller proportion of the total variance than when considering variability across all 10 observations (i.e., teacher‐led and child‐led activities). Compared to child‐led activities, teacher‐led morning circles are more consistent, routine activities that children experience daily, potentially yielding lower intra‐individual variability. However, within the teacher‐led activities, half of the variance was still explained by within‐person differences. This is consistent with finding only small associations between activity type and momentary attention and inhibitory control. In line with dynamic systems theory, these findings indicate that factors beyond activity type shape momentary self‐regulation (Holochwost et al. 2023; Perone et al. 2021). More research identifying other contributing factors is crucial for a comprehensive understanding of self‐regulation dynamics.

Surprisingly, in contrast to our fourth hypothesis, peer interactions were not significantly associated with children's self‐regulation skills. It is possible that the operationalization of peer interaction did not capture the most relevant aspects for self‐regulation. More precisely, as we only differentiated between the presence or absence of peer interactions, our measure did not reflect quality and type, which may be important factors for children's self‐regulation capacity (Alamos et al. 2022). Thus, a measure capturing the qualitative facets of peer interactions could provide more valuable insights regarding momentary social demands for children's self‐regulation. Another potential explanation for this finding is that peer interactions may become more relevant in middle childhood when interactions get increasingly complex (Finch et al. 2019).

The results revealed associations between children's individual characteristics age and gender with intra‐individual variability in attention and inhibitory control. Similar to previous studies focusing on cognitive and attention regulation in diverse age groups (Dirk and Schmiedek 2016; Janvier and Testu 2007; Williams et al. 2005), the present findings suggest that younger children showed more variability in attention control than older ones. However, the effect was small and not replicated in additional robustness analyses (i.e., coefficient of variation; Leonard et al. 2022). No age effects were found for inhibitory control. Regarding gender, we found no differences in children's attention control, but boys showed more variability in inhibitory control than girls. When looking at between‐level gender differences in inhibitory control, boys performed worse. Similar findings have been reported before (e.g., Matthews et al. 2009). A longitudinal study on self‐regulation trajectories also reported gender differences. The results indicated that girls show higher self‐regulation at age 3, but boys catch up by approximately 7 (Hosch et al. 2022). Similar gender asynchrony in inhibitory control development was reported in other studies (Grissom and Reyes 2019; Montroy et al. 2016). Thus, assuming regulation variability decreases as children develop (e.g., Williams et al. 2005), the higher variability observed in boys might reflect the findings regarding asynchronous development and is likely to stabilize with age. To gain further insight into sources of intra‐individual variability, future research should examine age and gender as potential moderators of the association between momentary self‐regulation and contextual demands.

### 4.3 Contribution to the Study of Self‐Regulation

Our findings support the theoretical notion of the dynamic nature of self‐regulation (e.g., Perone et al. 2021) by providing empirical evidence of substantial variability both within and between children as a function of contextual and individual factors. These results have important implications for self‐regulation research. While self‐regulation is predominantly assessed within a single context, the observed substantial intra‐individual variability across measurement occasions highlights the need for repeated assessments (Eberhart et al. 2023; Holochwost et al. 2023). Moving toward methodologies that incorporate repeated assessments may not always be feasible, but repeated measures can reduce the risk of bias and thus provide a more comprehensive understanding of children's self‐regulation capacities. The present findings indicate that self‐regulation not only can vary across different activity types but also across similar activity types. This pattern is consistent with dynamic systems theory which conceptualizes behavior as highly context‐sensitive and multi‐faceted (e.g., Perone et al. 2021). However, dynamic systems theory is a general developmental framework and not a self‐regulation model. Although existing self‐regulation models acknowledge the role of context of self‐regulation development (e.g., Bailey and Jones 2019; Blair and Ku 2022), the present findings, underscore the need for self‐regulation frameworks that more explicitly capture how immediate contextual demands shape momentary regulatory behavior (for example frameworks regarding executive functions, see: Holochwost et al. 2023; Perone et al. 2021). The foundational principles of the dynamic systems theory could provide the basis for a more dynamic, context‐dependent model of self‐regulation. Such a model would not only characterize regulatory processes across different timescales (i.e., from moments, over days, or years), but also generate theoretically grounded, testable assumptions about how individuals regulate their behavior in response to varying situational demands.

### 4.4 Limitations and Perspectives

The current study has several limitations. First, *t* = 10 measurement occasions are relatively few when using a DSEM framework, which could lead to convergence or power issues. However, simulation studies demonstrated that with *N* ≥ 200, *t* = 10 is sufficient for models that are not overly complex (Schultzberg and Muthén 2018). Moreover, in the present study, we could not assess self‐regulation across more measurement occasions due to feasibility reasons. This limits our ability to draw conclusions about self‐regulation fluctuations across shorter time intervals. Other methodologies, such as accelerometry seem promising for efficiently capturing children's self‐regulation skill across shorter intervals (Koepp and Gershoff 2024).

Second, our sample is limited to kindergarteners from the Swiss education system. As early childhood educational systems differ across countries, results may deviate in other contexts. Therefore, future studies should analyze variability in diverse early childhood educational contexts to gain a more comprehensive perspective. Further longitudinal research combining within‐ and between‐person approaches is needed to clarify how intra‐individual variability contributes to developmental change in self‐regulation.

Third, methodological aspects of the RRSM need to be considered. Across measurement occasions, children showed relatively high attention and inhibitory control, which aligns with other studies using the RRSM (Eberhart et al. 2024; McCoy et al. 2022). Moreover, all observations were categorized as either teacher‐ or child‐led. However, despite this differentiation, some uncontrolled variation remained within the same activity type as the activities varied in content. Although this variation reflects the dynamics of everyday regulatory demands, it constrains our ability to fully understand the sources of intra‐individual variability in self‐regulation. Future studies could collect more limited activity types, such as only book‐reading during teacher‐led and puzzling during child‐led activities. This could reduce the lack of experimental control inherent in observational measures in everyday contexts (Ahmed et al. 2021). Moreover, in the present study class membership was associated with children's self‐regulation variability. This emphasizes that class(room) differences beyond the teacher‐relationship might affect regulatory demands and individual regulatory variability. Thus, systematically accounting for contextual factors such as classroom chaos (Holochwost et al. 2023) or management (Martin et al. 2024), might add to a more comprehensive understanding of the sources of self‐regulation dynamics.

### 4.5 Conclusion

The present findings advance our understanding of intra‐individual variability in young children's self‐regulation highlighting that such fluctuations are typical for kindergarteners. Additionally, the findings highlight the need for considering how time‐varying contextual factors within the classroom shape momentary regulatory demands. The study extends the literature by providing initial evidence for the inter‐individual differences in children's self‐regulation variability and suggesting that individual as well as contextual factors may also contribute to these differences. Future research examining additional factors related to self‐regulation variability across developmental trajectories will be essential for further understanding the role of short‐term variability in self‐regulation development. Identifying relevant factors might ultimately inform more individualized and context‐sensitive approaches for supporting children's regulatory functioning.

## Author Contributions

**Nora Tilda Kunz**: methodology, writing – original draft, conceptualization, investigation, formal analysis, data curation, project administration, writing – review and editing. **Niamh Oeri**: conceptualization, writing – review and editing, supervision, project administration, investigation, writing – original draft.

## Funding Information

The authors have nothing to report.

## Conflicts of Interest

The authors declare no conflicts of interest.

## Supporting information




**Supporting Table 1A** *Overview of Children and Kindergarten Teachers per Kindergarten Class*
**Supporting Table 2A** *Short Version of the Coding Manual RRSM*
**Supporting Table 3A** *Multilevel Exploratory Factor Analysis Model Results*.
**Supporting Table 4A** *Exploratory Multilevel Location‐Scale Models for Attention and Inhibitory Control Separated for the Teacher‐led and the Child‐led Activities*
**Supporting Table 5A** *Multilevel Location‐Scale Models with Time‐Invariant (TSRC) and ‐Varying (Activity Type and Peer Interaction) Covariates for Attention and Inhibitory Control*
**Supporting Table 6A** *Multilevel Location‐Scale Model with all Covariates for Attention Control and Inhibitory Control in the Same Model*
**Supporting Figure 1A** *Multilevel Exploratory Factor Analysis Eigenvalue Plots*
**Supporting Figure 2A** *Path Diagram with Standardized Model Estimates for Attention Control*
**Supporting Figure 3A** *Path Diagram with Standardized Model Estimates for Inhibitory Control*

## Data Availability

Deidentified data and Mplus output files are available on the corresponding project on OSF (https://osf.io/qrc9e/overview?view_only=fb2180a3447d4f588d44ed8facae1cda).
