# Supplementary material for: Intra‐Individual Variability in Children's Self‐Regulation in Kindergarten Classrooms: An Observational Study
Source: Dev Sci. 2026 Aug 10;29(5):e70262. doi: 10.1111/desc.70262 (PMC13457985; doi:10.1111/desc.70262)
Supplement: Supplementary file 1 [file DESC-29-e70262-s001.docx]

**Appendix**

**Table 1A**

*Overview of Children and Kindergarten Teachers per Kindergarten Class*

| Kindergarten id | Nb. of kindergarten teachers | Total nb. of children | Nb. of participating children |
| --- | --- | --- | --- |
| 10 | 2 | 20 | 18 |
| 11 | 2 | 22 | 13 |
| 12 | 2 | 31 | 18 |
| 13 | 2 | 21 | 14 |
| 14 | 2 | 18 | 15 |
| 15 | 2 | 17 | 10 |
| 16 | 1 | 18 | 9 |
| 17 | 2 | 20 | 17 |
| 18 | 2 | 18 | 13 |
| 19 | 2 | 14 | 9 |
| 20 | 2 | 15 | 10 |
| 21 | 2 | 19 | 10 |
| 22 | 2 | 11 | 8 |
| 23 | 1 | 18 | 13 |
| 24 | 3 | 10 | 7 |
| 25 | 3 | 10 | 7 |
| 26 | 2 | 17 | 10 |

*Note.* Children were recruited from 17 public kindergartens in different (sub)urban areas of the German-speaking part of Switzerland.

**Table 2A**

*Short Version of the Coding Manual RRSM*

| **Item Nb.** | **Item** | **Behavioral-Anchor for the Item-Specific Skills** | |
| --- | --- | --- | --- |
| **1** | **Controls physical movement** |  | |
|  |  | *Does* | *Does not* |
|  | *Controls unintentional movements; ability to keep physical movements within the bounds of what is appropriate to the given activity. (Does not: Task completion not possible due to physical activity; disruptive intrusion into the personal space of others)* | - 4 = consistently controlled (leaves “bubble space” no more than one time; adjusts clothing or changes position calmly and controlled; moves in an intentional pattern) - 3 = controlled most of the time (leaves “bubble space” twice; plays restlessly with clothing/shoes; changes positions) | - 2 = Cannot sit still most of the time (uncoordinated movements; fidgets; runs across the classroom; leaves “bubble space” max. 1x) - 1 = Cannot sit still consistently, with exceptions (Inappropriate interaction with objects, consistently invading the “bubble space” of others and disturbing them, constant fidgeting) |
| **2** | **Attention control** |  | |
|  |  | *Does* | *Does not* |
|  | *Pays attention to the activity at hand* | - 4 = Is consistently attentive (looks at the current activity and responds physically to it; briefly looks away once: returns immediately to focus) - 3 = Is attentive most of the time (briefly looks away once: does not return immediately to focus; briefly looks away twice: returns immediately to focus) | - 2 = Is 1/2 of the time (= 1min 30s) inattentive (frequently looking away; takes a long time to return focus but attempts to re-engage; is day-dreaming or distracted) - 1 = Is (almost) consistently inattentive; 2min 30s or more (not present; constantly asking for assistance; is unmotivated; interrupts other children) |
| **3** | **Can shift attention appropriately within an activity or task*** |  | |
|  |  | *Does* | *Does not* |
|  | *(Code only when the observed situation requires that the child shifts his/her attention within an activity; e.g., a child draws a circle and then cuts it out, or switches from listening to answering a question in a circle)* | - 4 = Child can successfully shift (can be observed several times; changes roles, activities (without help)) - 3 = Shifting is successful most of the time (at least 1x observable; 1-2x delayed reaction) | - 2 = Shifting (focus) within the activity fails twice (e.g., delayed or no response when addressed directly), attention shifts between different speakers, fails 2/3 of the time - 1 = Gets 'stuck' in an activity, unable to transition to a new one, no response – e.g., completely drifts off in the circle (gaze turned away from the situation or fixed/absent) |
| **4** | **Maintains focus during or quickly returns to focus after a disruption/interruption^*^** |  | |
|  |  | *Does* | *Does not* |
|  | *(Code only if the flow of the activity is disrupted; if an acute and unexpected disruption/interruption takes place; e.g., when others invade personal space or directly address the child so they cannot focus on the activity/topic at hand; or a peer disrupts during circle time. 🡪 N/A if someone addresses the child in an open speaking atmosphere (relevant to the topic and not completely unrelated) without actively disrupting; overlapping “bubble spaces” of two children is not considered a disruption, as long as both sit calmly and do not seem disturbed by each other.* | - 4 = Maintains focus during a disruption/interruption, reacts and returns directly - 3 = Briefly loses focus after disruption/interruption but returns quickly | - 2 = Loses focus, takes long to return but visibly tries (e.g., frequent gaze shifting; mentally disengaged from the activity) - 1 = Does not return (does something else; joins the disruption) |
| **5** | **Can ignore minor/peripheral distractions during the activity** |  | |
|  |  | *Does* | *Does not* |
|  | *(Inconspicuous noise in the environment around the child (e.g., ambient noise) or chatter across the room (e.g., whispering by others during circle time): BASELINE ASSUMPTION: Does it, only coded if the child noticeably cannot.* | - 4 = Can ignore peripheral activities consistently, stays focused (performs the task concentrated; ignores outside sounds; distracted at most once briefly) - 3 = Can ignore peripheral activities most of the time; is temporarily slightly distracted multiple times (> 1x) (returns independently and quickly). | - 2 = Has difficulty to ignore peripheral activities or distracts themselves (>2x) completely drifting off: highly distracted; task is neglected; returning focus takes a long time - 1 = Pays more attention to any distraction than to their own task (seeks to distract other children or themselves; joins the distraction; looks at the person filming; permanently turns away from the task) |
| **6** | **Shows evidence of independent planning or monitoring^*^** |  | |
|  |  | *Does* | *Does not* |
|  | *(Code only when an intentional plan with at least one step is evident; e.g., searching for beads and threading them, building a tower; does not include responding to instructions from adults)* | - 4 = Makes at least one plan/concept and successfully acts on it (discusses the intention for an activity; monitors and reflects on own actions and corrects mistakes; attempts to prevent a predicted undesired event; 'preserves' a product, e.g., lets a painting dry; etc.) - 3 = Creates at least one plan/concept and is oriented towards it (implementation is partially successful; 50/50) | - 2 = Creates a plan but immediately forgets it or decides not to follow through (fails to recognize mistakes; plan is not helpful for this activity; inflexible in revising the plan; only works with direct support) - 1 = Throws themselves into the activity without thinking about what needs to be done (doesn't pause; unreflective; fails to reflect on their own mistakes) |
| **7** | **Shows evidence of listening^*^** |  | |
|  |  | *Does* | *Does not* |
|  | *Controls attention and follows along with another speaker in the moment (Evaluate less strictly in an open atmosphere during circle time, where many children talk over each other and during play!) – Code only if there are clear indications of listening. Does someone address the child? (If not: N/A)* | - 4 = Can focus attention almost consistently on the speaker and responds to what is said (acts responsively; participates in the conversation; body is oriented to the speaker) - 3 =Can focus attention most of the time on the speaker and responds (1x not) | - 2 = Does not listen most of the time (>2x) (is distracted by other things; turns or looks away; engages in an activity; misses auditory cues) - 1 = Almost consistently doesn’t listen (interrupts the speaker; efforts by the teacher to get the child to listen are ineffective) |
| **8** | **Remembers and enacts a series of instructions or completes a multi-step activity^*^** |  | |
|  |  | *Does* | *Does not* |
|  | *The item is about working memory and the active recall*  *of novel steps (Code only when documented accordingly by the observer; instruction consists of at least 3 steps)* | - 4 = Remembers instructions for a complex activity and can carry them out independently (may also help others; remembers rules of a new game; follows a task or worksheet) - 3 = Remembers instructions for a complex activity most of the time and can carry them out with the teacher's assistance | - 2 = Does not follow instructions most of the time (may partially start steps but gets lost; only follows instructions when each step is scaffolded by the teacher) - 1 = Does not follow instructions (Cannot start steps or completes only one step; unable to follow instructions even with support) |
| **9** | **Co-creates and/or follows group norms or rules when interacting with peers^*^** |  | |
|  |  | *Does* | *Does not* |
|  | *Peer-to-peer regulation (Code only when the activity requires collaboration or cooperation; interaction with peers)* | - 4 = Consistently establishes and follows group norms or rules in interactions with peers (follows explicit and implicit rules; accepts modifications; changes roles; plays peacefully side by side; shows a sense of fairness) - 3 = Establishes and follows group norms or rules in interactions with peers most of the time (fails once) | - 2 = Ignores rules and roles most of the time, with exceptions (≥2 times; only works with the teacher's support; does not follow rules but maintains some self-control and does not become disruptive) - 1 = Consistently ignores rules and roles (becomes upset or agitated during play; acts out; breaks things; pushes other children away) |
| **10** | **Follows classroom rules and routines independently** |  | |
|  |  | *Does* | *Does not* |
|  | *Children's ability to regulate themselves in service of classroom expectations/rules (follows independently): BASELINE-ASSUMPTION: Does (Coder can only observe “Does not”). As a 'guideline' for rule-breaking: If all children display this kind of behavior, the daily classroom routine will not work.* | - 4 = Consistently follows classroom rules and routines independently (self-regulates; e.g., pauses before speaking; follows instructions; completes tasks) - 3 = Follows classroom rules and routines most of the time independently (fails once) | - 2 = Does not follow classroom routines / rules most of the time (=/> 2x; needs routines modeled by the teacher or peers; can self-regulate temporarily after a verbal prompt, but then gets distracted during the routine and does not return) - 1 = Consistently does not follow classroom routines/rules (directly violates rules; requires frequent prompting) |
| **11** | **Transition*** |  | |
|  |  | *Does* | *Does not* |
|  | *(not filmed)* | - Always N/A! | - Always N/A! |
| **12** | **Inhibits inappropriate or automatic responses and enacts appropriate responses** |  | |
|  |  | *Does* | *Does not* |
|  | *In-the-moment impulse control (control of automatic behavioral reactions; Does the child behave appropriately in the situation?) BASELINE-ASSUMPTION: Does (coder can only observe “Does not”). SR = self-regulation.* | - 4 = Consistently inhibits inappropriate or automatic reactions (uses appropriate skills to achieve goals; successfully engages in SR-games; does not blurt out; raises hand) - 3 = Inhibits inappropriate or automatic reactions most of the time (fails once) | - 2 = Acts impulsively most of the time (≥2 times; tries to impose their will and just does it; interrupts; cannot successfully do SR-games; rushes off to the next activity before the teacher signals a transition) - 1 = Consistently acts impulsively |
| **13** | **Modulates emotional arousal or maintains appropriate level of emotional arousal in response to classroom expectations** |  | |
|  |  | *Does* | *Does not* |
|  | *E = emotion:BASELINE-ASSUMPTION: Does (coder can only observe “Does not”: Considering the overall goals of the classroom ).* | - 4 = Consistently regulates E appropriately to the context, and maintains an appropriate level of E arousal in response to classroom expectations (E matches the feelings of others) - 3 = Regulates E appropriately to the context, and maintains an appropriate level of E arousal in response to classroom expectations most of the time (fails once) | - 2 = E-Regulation is inappropriate most of the time (≥2 times; E deviates from the arousal of their peers, with much more or much less expression) - 1 = E-Regulation is consistently inappropriate (unable to calm down when angry or upset; emotional state is disruptive and carries over to other activities; emotional state interferes with their ability to complete an activity) |
| **14** | **Regulates behavior in the face of own emotional arousal^*^** |  | |
|  |  | *Does* | *Does not* |
|  | *E = emotion: Code only during acute emotional situations/exceptionally emotionally arousing events, when obvious emotional arousal is shown. (If no such arousal is present: N/A)* | - 4 = Consistently regulates behavior during acute emotional situations (copes with conflict/frustration/disappointment; responds appropriately to the context; works toward the classroom goals even while emotionally aroused) - 3 = Regulates behavior during acute emotional situations most of the time (fails once) | - 2 = Responds inappropriately most of the time to an emotional situation (≥2 times; reacts emotionally and struggles to get over the situation; expresses E in inappropriate ways such as whining, exaggerated joy, etc.) - 1 = Consistently responds inappropriately to emotional situations (…, struggles to get over the situation even with support from the teacher or other children; upcoming tasks can hardly be managed) |
| **15** | **Is able to wait for something^*^** |  | |
|  |  | *Does* | *Does not* |
|  | *Sustained inhibitory control (Code when the situation clearly demands waiting: e.g., CIRCLE: pause, wait until the teacher shows the book. PLAY: turn to share material; reaction of others: when the child addresses someone, and if there is no response, they nudge, become louder, etc.)* | - 4 = Can consistently wait for something when required (e.g., to speak; for materials; to start an activity; etc.), remains calm and shows patience, can hold back - 3 = Can wait for something most of the time, when required (fails once) | - 2 = Does not wait most of the time (≥2 times; talks over partner; is impatient; becomes angry; disrupts others; avoids waiting, but makes an effort and manages to some extent) - 1 = Consistently does not wait (cannot hold back and shows no effort; does not wait turn) |
| **16** | **Shows evidence of ability to solve and cope with social dilemmas and conflicts with peers^*^** |  | |
|  |  | *Does* | *Does not* |
|  | *Shows signs of social problem-solving (Code only when unusual behavior is exhibited by a peer – not when the target child behaves inappropriately towards others, but when they need to react to it, e.g., when someone wants the child’s toy)* | - 4 = Consistently shows signs of the ability to handle the situation (responds appropriately to unusual behavior from peers (if other child does not meet Item 9); empathizes, negotiates; has a sense of fairness, applies problem-solving strategies; avoids potential conflict/ignores it) - 3 = Handles the situation most of the time (fails once) | - 2 = Cannot handle the situation most of the time (≥2 times; gets upset is unable to calm down enough to employ problem-solving skills; runs away during disagreement/dilemma; tries to impose their will; becomes physically or verbally aggressive) - 1 = Consistently cannot handle it (…) |

*Note.* The Regulation-Related Skills Measure was developed by Dr. McCoy and the RRSM team. For additional information, see <https://rrsm.hsites.harvard.edu/>; McCoy, D. C., Koepp, A. E., Jones, S. M., Bodrova, E., Leong, D. J., & Deaver, A. H. (2022). An observational approach for exploring variability in young children’s regulation-related skills within classroom contexts. *Developmental Science, 25*(6). <https://doi.org/10.1111/desc.13250>. The observation and scoring guide was translated and edited into (Swiss-)German. Some examples of the items in the observation guide were adapted to match the Swiss kindergarten classroom context (examples were screened by a Swiss kindergarten teacher). This coding sheet is a back-translation from (Swiss-)German into English.

**Table 3A**

*Multilevel Exploratory Factor Analysis Model Results.*

| Number of Factors Within and Between | RMSEA | TLI |
| --- | --- | --- |
| One factor within and one factor between | .165 | .756 |
| Two factors within and one factor between | .059 | .969 |
| Three factors within and one factor between | .055 | .973 |
| One factor within and two factors between | .183 | .700 |
| **Two factors within and two factors between** | **.052** | **.976** |
| Three factors within and two factors between | .024 | .995 |
| One factor within and three factors between | .196 | .657 |
| Two factors within and three factors between | .057 | .971 |
| Three factors within and three factors between | .015 | .998 |

*Note.* Model fit information for the multilevel exploratory factor analysis (MEFA) with the final included RRSM items. RMSEA = root-mean-square error of approximation. TLI = Tucker-Lewis-Index.

**Table 4A**

*Exploratory Multilevel Location-Scale Models for Attention and Inhibitory Control Separated for the Teacher-led and the Child-led Activities*

| **H1 H2 AC** | | | | | |  | **H1 H2 IC** | | | | | |
| --- | --- | --- | --- | --- | --- | --- | --- | --- | --- | --- | --- | --- |
|  | **Model teacher-led activity** | |  | **Model child-led activity** | |  |  | **Model teacher-led activity** | |  | **Model child-led activity** | |
| Effect | Est. | 95% CI |  | Est. | 95% CI |  | Effect | Est. | 95% CI |  | Est. | 95% CI |
| Intercept AC | 3.54 | [3.48 / 3.60] |  | 3.57 | [3.53 / 3.60] |  | Intercept IC | 3.81 | [3.76 / 3.84] |  | 3.97 | [3.96 / 3.98] |
| Intercept LogV | -2.09 | [-2.31 / -1.89] |  | -2.22 | [-2.40 / -2.05] |  | Intercept LogV | -2.76 | [-3.05 / -2.48] |  | -3.58 | [-3.88 / -3.31] |
| Variance AC | 0.11 | [0.07 / 0.16] |  | 0.03 | [0.02 / 0.05] |  | Variance IC | 0.03 | [0.02 / 0.04] |  | 0.00 | [0.00 / 0.00] |
| Variance LogV | 1.29 | [0.85 / 1.83] |  | 0.89 | [0.59 / 1.24] |  | Variance LogV | 3.43 | [2.74 / 4.27] |  | 3.79 | [3.08 / 4.71] |
| Nb. of observations | 943 | |  | 963 | |  |  | 943 | |  | 963 | |

*Note.* Exploratory multilevel location-scale models with only the teacher-led or the child-led activities, with attention control (AC) and inhibitory control (IC) as the dependent variables. Est. = Unstandardized posterior median. CI = Credibility interval.

**Table 5A**

*Multilevel Location-Scale Models with Time-Invariant (TSRC) and -Varying (Activity Type and Peer Interaction) Covariates for Attention and Inhibitory Control*

| **Model with covariates AC** | | |  | **Model with covariates IC** | | |
| --- | --- | --- | --- | --- | --- | --- |
| Effect | Est | 95% CI |  | Effect | Est. | 95% CI |
| Intercept AC | 3.56 | [3.53 / 3.59] |  | Intercept IC | 3.85 | [3.82 / 3.87] |
| Intercept activity type | 0.51 | [0.48 / 0.53] |  | Intercept activity type | 0.51 | [0.48 / 0.53] |
| Intercept peer interaction | 0.37 | [0.34 / 0.39] |  | Intercept peer interaction | 0.37 | [0.34 / 0.39] |
| Intercept TSR closeness | -0.00 | [-0.10 / 0.10] |  | Intercept TSR closeness | -0.00 | [-0.10 / 0.10] |
| Intercept LogV | -1.75 | [-2.13 / -1.35] |  | Intercept LogV | -2.66 | [-3.41 / -1.91] |
| AC on activity type | -0.09 | [-0.14 / -0.04] |  | IC on activity type | 0.03 | [0.01 / 0.04] |
| AC on peer interaction | 0.04 | [-0.01 / 0.10] |  | IC on peer interaction | -0.01 | [-0.02 / 0.01] |
| LogV on TSR closeness | -0.34 | [-0.55 / -0.11] |  | LogV on TSR closeness | -0.17 | [-0.59 / 0.28] |
| LogV on kiga_11 | -0.58 | [-1.21 / 0.09] |  | LogV on kiga_11 | -0.37 | [-1.63 / 0.91] |
| LogV on kiga_12 | -0.02 | [-0.55 / 0.55] |  | LogV on kiga_12 | -1.14 | [-2.23 / -0.05] |
| LogV on kiga_13 | 0.02 | [-0.56 / 0.60] |  | LogV on kiga_13 | -0.95 | [-2.13 / 0.20] |
| LogV on kiga_14 | -0.88 | [ -1.47 / -0.32] |  | LogV on kiga_14 | 0.14 | [-1.01 / 1.25] |
| LogV on kiga_15 | -0.32 | [-1.01 / 0.30] |  | LogV on kiga_15 | 0.63 | [-0.71 / 1.88] |
| LogV on kiga_16 | 0.29 | [-0.36 / 0.95] |  | LogV on kiga_16 | -0.68 | [-1.94 / 0.63] |
| LogV on kiga_17 | 0.33 | [-0.21 / 0.88] |  | LogV on kiga_17 | 0.67 | [-0.38 / 1.75] |
| LogV on kiga_18 | 0.12 | [-0.46 / 0.68] |  | LogV on kiga_18 | 0.21 | [-0.98 / 1.34] |
| LogV on kiga_19 | -0.01 | [-0.63 / 0.69] |  | LogV on kiga_19 | 0.08 | [- 1.22 / 1.42] |
| LogV on kiga_20 | -0.01 | [-0.65 / 0.63] |  | LogV on kiga_20 | 0.78 | [-0.54 / 2.01] |
| LogV on kiga_21 | -0.11 | [-0.76 / 0.54] |  | LogV on kiga_21 | -1.74 | [- 2.99 / -0.47] |
| LogV on kiga_22 | -0.52 | [-1.23 / 0.15] |  | LogV on kiga_22 | -0.28 | [-1.72 / 1.04] |
| LogV on kiga_23 | 1.04 | [0.46 / 1.63] |  | LogV on kiga_23 | -0.12 | [-1.23 / 1.02] |
| LogV on kiga_24 | -0.02 | [-0.77 / 0.70] |  | LogV on kiga_24 | 0.83 | [-0.59 / 2.21] |
| LogV on kiga_25 | 1.12 | [0.40 / 1.84] |  | LogV on kiga_25 | 0.92 | [-0.54 / 2.34] |
| LogV on kiga_26 | -0.37 | [-0.99 / 0.26] |  | LogV on kiga_26 | 0.12 | [-1.13 / 1.42] |
| Variance AC | 0.03 | [0.02 / 0.04] |  | Variance IC | 0.01 | [0.01 / 0.02] |
| Variance activity type | 0.00 | [0.00 / 0.00] |  | Variance activity type | 0.00 | [0.00 / 0.00] |
| Variance peer interaction | 0.01 | [0.00 / 0.02] |  | Variance peer interaction | 0.01 | [0.01 / 0.02] |
| Variance TSR closeness | 0.49 | [0.40 / 0.60] |  | Variance TSR closeness | 0.49 | [0.40 / 0.61] |
| Variance LogV | 0.41 | [0.29 / 0.59] |  | Variance LogV | 2.41 | [1.94 / 3.05] |
| R^2^ within | 1.0% |  |  |  | 0.8% |  |
| R^2^ between | 45.5% |  |  |  | 23.4% |  |

*Note.* Estimates (Unstandardized posterior median) and 95% credible intervals (CI) for the multilevel location-scale models with attention control (AC) and inhibitory control (IC) as dependent variables and with the time-invariant teacher-student relationship closeness (TSRC), and time-varying covariates activity type (AT), and peer interaction (PI). Weekday (WD) was added at the within-level, while age and gender were added at the between-level. Kiga_11 to kiga_26 are dummy-coded variables representing class membership at the between-level. *N* = 1906.

**Table 6A**

*Multilevel Location-Scale Model with all Covariates for Attention Control and Inhibitory Control in the Same Model*

| **Full Model** | | |
| --- | --- | --- |
| Effect | Est | 95% CI |
| Intercept AC | 3.56 | [3.53 / 3.59] |
| Intercept IC | 3.85 | [3.82 / 3.87] |
| Intercept activity type | 0.51 | [0.48 / 0.53] |
| Intercept peer interaction | 0.37 | [0.34 / 0.39] |
| Intercept weekday | 2.90 | [2.83 / 2.98] |
| Intercept TSR closeness | -0.00 | [-0.10 / 0.10] |
| Intercept age | 0.01 | [-1.05 / 1.10] |
| Intercept gender | 0.49 | [0.42 / 0.56] |
| Intercept LogVAC | -1.71 | [-2.11 / -1.29] |
| Intercept LogVIC | -2.16 | [-2.90 / -1.42] |
| AC on activity type | -0.09 | [-0.14 / -0.04] |
| AC on peer interaction | 0.04 | [-0.01 / 0.10] |
| AC on weekday | 0.01 | [-0.00 / 0.02] |
| IC on activity type | 0.03 | [0.01 / 0.04] |
| IC on peer interaction | -0.01 | [-0.02 / 0.01] |
| IC on weekday | -0.00 | [-0.00 / 0.00] |
| LogVAC on TSR closeness | -0.30 | [-0.52 / -0.06] |
| LogVAC on age | -0.02 | [-0.04 / -0.01] |
| LogVAC on gender | -0.07 | [-0.32 / 0.18] |
| LogVAC on kiga_11 | -0.59 | [-1.23 / 0.10] |
| LogVAC on kiga_12 | -0.01 | [-0.55 / 0.52] |
| LogVAC on kiga_13 | -0.03 | [-0.63 / 0.56] |
| LogVAC on kiga_14 | -0.84 | [ -1.43 / -0.28] |
| LogVAC on kiga_15 | -0.30 | [-0.96 / 0.35] |
| LogVAC on kiga_16 | 0.19 | [-0.44 / 0.85] |
| LogVAC on kiga_17 | 0.30 | [-0.23 / 0.85] |
| LogVAC on kiga_18 | 0.11 | [-0.48 / 0.70] |
| LogVAC on kiga_19 | -0.01 | [-0.68 / 0.63] |
| LogVAC on kiga_20 | -0.02 | [-0.65 / 0.61] |
| LogVAC on kiga_21 | -0.17 | [-0.82 / 0.48] |
| LogVAC on kiga_22 | -0.54 | [-1.21 / 0.16] |
| LogVAC on kiga_23 | 1.07 | [0.49 / 1.68] |
| LogVAC on kiga_24 | 0.06 | [-0.66 / 0.83] |
| LogVAC on kiga_25 | 1.24 | [0.55 / 1.97] |
| LogVAC on kiga_26 | -0.39 | [-1.00 / 0.22] |
| LogVIC on TSR closeness | 0.20 | [-0.26 / 0.64] |
| LogVIC on age | 0.00 | [-0.03 / 0.03] |
| LogVIC on gender | -1.00 | [-1.48 / -0.52] |
| LogVIC on kiga_11 | 0.06 | [-1.21 / 1.33] |
| LogVIC on kiga_12 | -1.24 | [-2.27 / -0.22] |
| LogVIC on kiga_13 | -0.80 | [-1.85 / 0.29] |
| LogVIC on kiga_14 | 0.16 | [-0.91 / 1.20] |
| LogVIC on kiga_15 | 0.83 | [-0.48 / 2.11] |
| LogVIC on kiga_16 | -0.85 | [-2.04 / 0.48] |
| LogVIC on kiga_17 | 0.48 | [-0.63 / 1.52] |
| LogVIC on kiga_18 | 0.32 | [-0.82 / 1.49] |
| LogVIC on kiga_19 | -0.06 | [- 1.27 / 1.23] |
| LogVIC on kiga_20 | 0.85 | [-0.31 / 2.09] |
| LogVIC on kiga_21 | -2.02 | [- 3.28 / -0.88] |
| LogVIC on kiga_22 | -0.43 | [-1.74 / 0.92] |
| LogVIC on kiga_23 | -0.07 | [-1.16 / 1.04] |
| LogVIC on kiga_24 | 0.76 | [-0.60 / 2.17] |
| LogVIC on kiga_25 | 0.83 | [-0.54 / 2.23] |
| LogVIC on kiga_26 | -0.04 | [-1.23 / 1.21] |
| Variance AC | 0.03 | [0.02 / 0.04] |
| Variance IC | 0.01 | [0.01 / 0.02] |
| Variance activity type | 0.00 | [0.00 / 0.00] |
| Variance peer interaction | 0.01 | [0.01 / 0.02] |
| Variance weekday | 0.06 | [0.01 / 0.13] |
| Variance TSR closeness | 0.51 | [0.42 / 0.63] |
| Variance age | 60.13 | [49.68 / 74.56] |
| Variance gender | 0.28 | [0.23 / 0.34] |
| Variance LogVAC | 0.39 | [0.28 / 0.55] |
| Variance LogVIC | 2.21 | [1.78 / 2.79] |
| AC R^2^ within | 1.2% |  |
| IC R^2^ within | 0.9% |  |
| AC R^2^ between | 49.6% |  |
| IC R^2^ between | 32.1% |  |

*Note.* Estimates (Unstandardized posterior median) and 95% credible intervals (CI) for the multilevel location-scale models with attention control (AC) and inhibitory control (IC) as dependent variables and with the time-invariant teacher-student relationship closeness (TSRC), and time-varying covariates activity type (AT), and peer interaction (PI). Weekday (WD) was added at the within-level, while age and gender were added at the between-level. Kiga_11 to kiga_26 are dummy-coded variables representing class membership at the between level. *N* = 1906.

**Figure 1A**

*Multilevel Exploratory Factor Analysis Eigenvalue Plots*

*Note.* Eigenvalue plots for the multilevel exploratory factor analysis (MEFA) with the final included RRSM items. The plot on the top represents the solution on the within-level and the plot on the bottom the solution on the between-level.

**Figure 2A**

*Path Diagram with Standardized Model Estimates for Attention Control*

*Note.* Diagrams are simplified, not all parameters reported. Rectangles represent observed measures, circles represent latent variables, triangles represent intercepts. Log σ = within-person residual variance, AC = attention control. TSR= teacher-student relationship. Class m. = class membership. 𝛼 = intercept AC. Significant paths are drawn in solid lines.

**Figure 3A**

*Path Diagram with Standardized Model Estimates for Inhibitory Control*

*Note.* Diagrams are simplified, not all parameters reported. Rectangles represent observed measures, circles represent latent variables, triangles represent intercepts. Log σ = within-level person variance, IC = inhibitory control. TSR= teacher-student relationship. Class m. = class membership. 𝛼 = intercept IC. Significant paths are drawn in solid lines.
